# Fermented Oyster Extract Promotes Insulin-Like Growth Factor-1-Mediated Osteogenesis and Growth Rate

**DOI:** 10.3390/md18090472

**Published:** 2020-09-18

**Authors:** Ilandarage Menu Neelaka Molagoda, Jayasingha Arachchige Chathuranga Chanaka Jayasingha, Yung Hyun Choi, Eui Kyun Park, You-Jin Jeon, Bae-Jin Lee, Gi-Young Kim

**Affiliations:** 1 Department of Marine Life Science, Jeju National University, Jeju 63243, Korea; neelakagm2012@gmail.com (I.M.N.M.); chanakajayasinghe7@gmail.com (J.A.C.C.J.); youjinj@jejunu.ac.kr (Y.-J.J.); 2 Department of Biochemistry, College of Oriental Medicine, Dong-Eui University, Busan 47227, Korea; choiyh@deu.ac.kr; 3 Department of Oral Pathology and Regenerative Medicine, School of Dentistry, Institute for Hard Tissue and Biotooth Regeneration, Kyungpook National University, Daegu 41940, Korea; epark@knu.ac.kr; 4 Marine Bioprocess Co., Ltd., Busan 46048, Korea; hansola82@hanmail.net

**Keywords:** *Crassostrea gigas*, growth performance, osteogenesis, IGF-1, GSK-3β, RUNX2

## Abstract

Fermented oyster (*Crassostrea gigas*) extract (FO) prevents ovariectomy-induced osteoporosis by inhibiting osteoclastogenesis and activating osteogenesis. However, the molecular mechanisms underlying FO-mediated bone formation and growth rate are unclear. In the current study, we found that FO significantly upregulated the expression of growth-promoting genes in zebrafish larvae including insulin-like growth factor 1 (*zigf-1*), insulin-like growth factor binding protein 3 (*zigfbp-3*), growth hormone-1 (*zgh-1*), growth hormone receptor-1 (*zghr-1*), growth hormone receptor alpha (*zghra*), glucokinase (*zgck*), and cholecystokinin (*zccka*). In addition, zebrafish larvae treated with 100 μg/mL FO increased in total body length (3.89 ± 0.13 mm) at 12 days post fertilization (dpf) compared to untreated larvae (3.69 ± 0.02 mm); this effect was comparable to that of the β-glycerophosphate-treated zebrafish larvae (4.00 ± 0.02 mm). Furthermore, FO time- and dose-dependently increased the extracellular release of IGF-1 from preosteoblast MC3T3-E1 cells, which was accompanied by high expression of *IGF-1*. Pharmacological inhibition of IGF-1 receptor (IGF-1R) using picropodophyllin (PPP) significantly reduced FO-mediated vertebrae formation (from 9.19 ± 0.31 to 5.53 ± 0.35) and growth performance (from 3.91 ± 0.02 to 3.69 ± 0.01 mm) in zebrafish larvae at 9 dpf. Similarly, PPP significantly decreased FO-induced calcium deposition in MC3T3-E1 cells by inhibiting GSK-3β phosphorylation at Ser9. Additionally, DOI hydrochloride, a potent stabilizer of GSK-3β, reduced FO-induced nuclear translocation of RUNX2. Transient knockdown of *IGF-1Rα*/*β* using specific silencing RNA also resulted in a significant decrease in calcium deposition and reduction in GSK-3β phosphorylation at Ser9 in MC3T3-E1 cells. Altogether, these results indicate that FO increased phosphorylated GSK-3β at Ser9 by activating the autocrine IGF-1-mediated IGF-1R signaling pathway, thereby promoting osteogenesis and growth performance. Therefore, FO is a potential nutritional supplement for bone formation and growth.

## 1. Introduction

Insulin-like growth foctor-1 (IGF-1) is a 70-amino acid polypeptide hormone similar to insulin in molecular structure and is present in both humans and rodents [1]. Despite being involved in the mediation of normal physiological functions such as tissue growth and development [2], lipid metabolism [3], anti-inflammatory responses [4], and anabolic functions [5], IGF-1 is also crucial for osteoblast differentiation, bone remodeling, and growth performance [6]. Although IGF-1 is mainly produced by the liver, all other tissues also produce a considerable amount of IGF-1 [7]. IGF-1 is fragile and unstable during systemic circulation; hence, it normally binds with specific IGF-binding proteins (IGFBPs) to achieve a prolonged half-life, thus enabling its transport from the blood circulation to the distinct tissues [8]. Many studies have demonstrated that IGF-1 strongly binds to IGFBPs with higher specificity and affinity than to IGF-1 receptor (IGF-1R), thereby weakening the ligation of IGF-1 to IGF-1R [9,10]. In this regard, IGFBP-specific proteases such as pregnancy-associated plasma protein A (PAPP-A) and PAPP-A_2_ cleave IGFBPs to liberate IGF-1 from IGF-1/IGFBP complex, leading to increased binding affinity of IGF-1 to IGF-1R [11].

IGF-1R differs from other receptor families due to the existence of disulfide-linked dimers (two α and two β subunits) and the requirement of domain rearrangement to initiate intracellular signaling [12]. The ligation of IGF-1 to the α-subunit of IGF-1R induces conformational changes in the receptor, leading to autophosphorylation of the β-subunit in IGF-1R and activation of the downstream cellular signaling pathways [13]. Under normal physiological conditions, stimulation of the IGF-1R signaling pathway inactivates glycogen synthase kinase-3β (GSK-3β) through phosphorylation at Ser9 and thus is mediated by activation of the phosphoinositide 3-kinase (PI3K) and protein kinase B (also known as Akt) signaling pathways [14]. This consequently releases β-catenin from the destructive complex and thereby enables β-catenin-related transcriptional activation for osteogenesis [15]. Additionally, Kugimiya et al. demonstrated that heterozygous GSK-3β-deficient mice showed enhanced bone formation mediated by the upregulation of runt-related transcription factor 2 (RUNX2) transcriptional activities [16]. Therefore, IGF-1 may activate β-catenin and RUNX2 by inhibiting GSK-3β and enhance osteogenesis-mediated growth performance.

In our previous study, the composition of fermented oyster (*Crassostrea gigas*) extract (FO) was characterized, and we reported that FO induced osteoblast differentiation and bone formation by activating the β-catenin signaling pathway [17]. Additionally, our research team demonstrated that FO prevented ovariectomy-induced bone loss by inhibiting osteoclastogenesis [18,19]. However, it is unclear whether FO positively regulated growth performance concomitantly with bone formation. In the current study, we aimed to elucidate the mechanism through which FO potentially stimulates osteogenesis-mediated growth performance. Our data revealed that FO-mediated bone formation and growth performance were regulated by activation of the IGF-1R signaling pathway and concomitant expression of autocrine IGF-1, which induced GSK-3β phosphorylation at Ser9, activated RUNX2, and ultimately resulted in osteogenesis and improved growth performance.

## 2. Results

### 2.1. FO Promotes Total Growth Rate in Zebrafish Larvae

**Figure 1:**
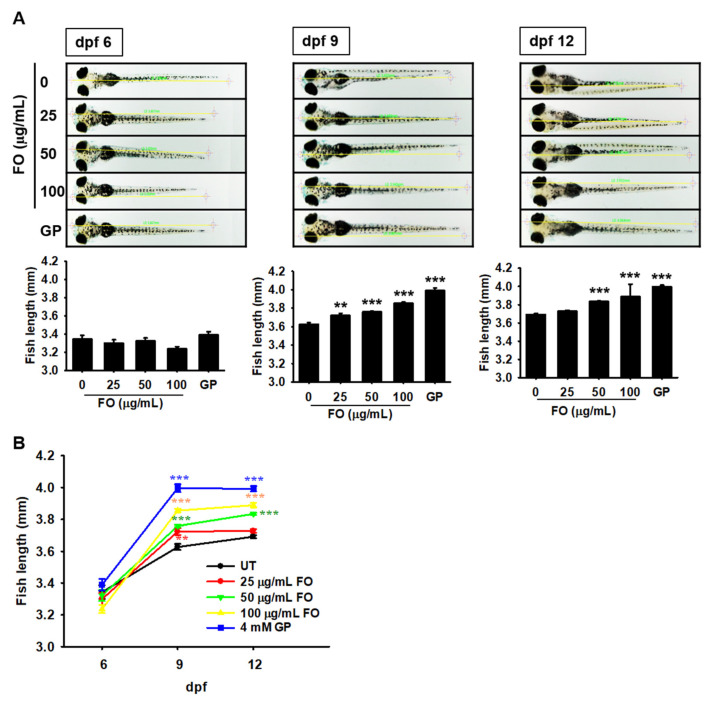
FO promotes the growth of zebrafish larvae. Zebrafish larvae (*n* = 30) at 3 days post fertilization (dpf) were treated with the indicated concentrations of FO (0–100 µg/mL). The total body lengths were measured at (**A**) 6, 9, and 12 dpf using an Olympus microscope (×4). β-glycerophosphate (GP) at 4 mM was used as a positive control. (**B**) Graphical representation of the total body length at each dpf. Significant differences among the groups were determined using one-way ANOVA followed by Bonferroni correction. All data are presented as mean ± SEM (001 ** *p* < 0.01 and *** *p* < 0.001 vs. untreated zebrafish larvae). FO, fermented oyster (*C. gigas*) extract; UT, untreated.

To evaluate the effect of FO on growth rate, zebrafish larvae were treated with FO (0–100 μg/mL) at 3 dpf, and the total body lengths were measured at 6, 9, and 12 dpf. By 6 dpf, no change in the total body length was observed, regardless of FO treatment. From 6 to 9 dpf, the body length of the untreated zebrafish larvae significantly increased from 3.34 ± 0.04 to 3.63 ± 0.02 mm, which was sustained till 12 dpf (3.69 ± 0.02 mm). At 9 dpf, FO-treated zebrafish larvae showed a significant increase in total body length compared to the untreated zebrafish larvae (Figure 1A). As shown in Figure 1B, treatment with FO increased the total body length to 3.72 ± 0.01, 3.76 ± 0.01, and 3.86 ± 0.01 mm at 25, 50, and 100 µg/mL FO, respectively, at 9 dpf. GP-treated zebrafish larvae showed the most prominent total body length (3.99 ± 0.02 mm) at 9 dpf. Unexpectedly, 25 (3.73 ± 0.02) and 100 µg/mL (3.89 ± 0.13 mm) of FO did not show any significant improvement at 12 dpf compared with those at 9 dpf. Moreover, treatment with 50 µg/mL FO resulted in a significant increase in total body length at 12 dpf (3.83 ± 0.01 mm) and the effect was comparable to that of the GP-treated larvae (4.00 ± 0.02 mm). Altogether, these results indicate that FO is a potent stimulator of growth.

### 2.2. FO Upregulates the Expression of Growth-Promoting Genes

**Figure 2:**
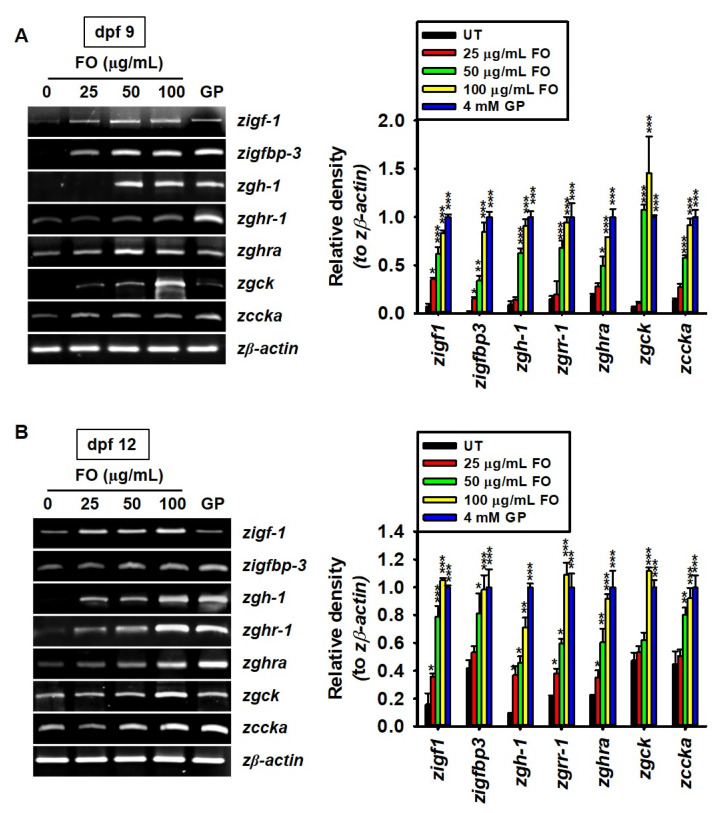
FO upregulates the expression of growth-promoting genes. Zebrafish larvae (*n* = 30) at 3 days post fertilization (dpf) were treated with the indicated concentrations of FO (0–100 µg/mL). β-glycerophosphate (GP) at 4 mM was used as a positive control. (**A**,**B**) Total mRNA was extracted at 9 (**A**) and 12 (**B**) dpf, and reverse transcription polymerase chain reaction was performed to measure the expression of insulin-like growth factor 1 (*zigf-1)*, insulin-like growth factor binding protein 3 (*zigfbp-3*), growth hormone 1 (*zgh-1*), growth hormone receptor 1 (*zghr-1*), growth hormone receptor alpha (*zghra*), glucokinase (*zgck*), and cholecystokinin (*zccka*). β-actin was used as an internal control. The relative density was calculated using ImageJ software. Significant differences among the groups were determined using one-way ANOVA followed by Bonferroni correction. All data are presented as mean ± SEM (* *p* < 0.05, ** *p* < 0.01, and *** *p* < 0.001 vs. untreated zebrafish larvae). FO, fermented oyster (*C. gigas*) extract; UT, untreated.

FO considerably increased the growth rate of zebrafish larvae; hence, we verified whether it can positively regulate the expression of growth-promoting genes such as *zigf-1*, *zigfbp-3*, *zgh-1*, *zghr-1*, *zghra*, *zgck*, and *zccka* in zebrafish larvae. At 9 dpf, we found that the expression of *zigf-1* was significantly increased in FO-treated larvae, even at 25 µg/mL (relative density compared to GP treatment: 0.35 ± 0.02), than in the untreated larvae (0.07 ± 0.01), and higher concentrations of FO further enhanced the expression of *zigf-1* (Figure 2A). In 9 dpf zebrafish larvae, the relative expression of *zigfbp-3* (0.34 ± 0.05 and 0.84 ± 0.09), *zgh-1* (0.63 ± 0.05 and 0.91 ± 0.07), *zghr-1* (0.68 ± 0.07 and 0.94 ± 0.06), *zghra* (0.49 ± 0.09 and 0.79 ± 0.08), *zgck* (1.08 ± 0.05 and 1.45 ± 0.37), and *zccka* (0.58 ± 0.03 and 0.91 ± 0.07) significantly increased with 50 and 100 µg/mL FO, respectively, which was comparable to those with GP treatment. The same genes were further analyzed in 12 dpf zebrafish larvae. *zigf-1*, *zgh-1*, *zghr-1*, and *zghra* were significantly expressed in 25 µg/mL FO-treated zebrafish larvae than in the untreated larvae (Figure 2B). However, the expression of *zigfbp-3*, *zgck*, and *zccka* became significant ≥ at 50 µg/mL, compared to that observed in the untreated conditions. The gene expression under the GP-treated conditions was comparable to that at 100 µg/mL of FO. Altogether, these results indicate that FO improved growth performance by upregulating the expression of growth-promoting genes.

### 2.3. FO Promotes the Release of IGF-1 in MC3T3-E1 Cells by Transactivating Its Gene Expression

**Figure 3:**
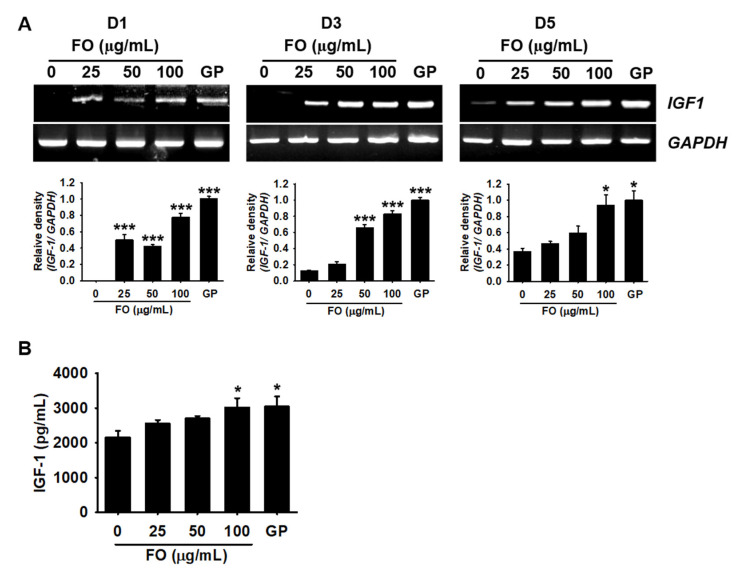
FO upregulates IGF-1 expression in preosteoblast MC3T3-E1 cells. (**A**) MC3T3-E1 cells were seeded at a density of 2000 cells/cm^2^ and treated with the indicated concentrations of FO (0–100 µg/mL) for 5 days. β-glycerophosphate (GP) at 2 mM was used as a positive control. Total mRNA was extracted at days 1, 3, and 5 after treatment with FO. Reverse transcription polymerase chain reaction was performed to determine the expression level of *IGF-1*. *GAPDH* was used as an internal control. The expression of *IGF-1* relative to that of *GAPDH* level was determined (bottom). (**B**) In a parallel experiment, the cells were treated with FO or GP for 3 days. Cell culture media were collected, and colorimetric ELISA was performed to quantify the levels of extracellular IGF-1. Significant differences among the groups were determined using one-way ANOVA followed by Bonferroni correction. All data are presented as mean ± SEM (* *p* < 0.05 and *** *p* < 0.001 vs. untreated MC3T3-E1 cells). FO, fermented oyster (*C. gigas*) extract.

IGF-1 is known to promote bone formation and growth performance; hence, we investigated whether FO could increase the extracellular release of IGF-1. MC3T3-E1 cells were treated with FO for 5 days, and the expression of *IGF-1* was determined on days 1, 3, and 5. As shown in Figure 3A, the expression of *IGF-1* was significantly higher at day 1 in FO-treated cells (relative density compared to GP treatment: 0.49 ± 0.07, 0.42 ± 0.02, and 0.78 ± 0.05 at 25, 50, and 100 µg/mL FO, respectively) than in the untreated cells. From day 3, the untreated cells began to slightly express *IGF-1* and weakly sustained the expression till day 5. At day 3, FO increased the expression of IGF-1 (0.21± 0.03, 0.66 ± 0.04, and 0.83 ± 0.04 at 25, 50, and 100 µg/mL, respectively) in a dose-dependent manner, which was sustained till day 5. Next, we evaluated the extracellular release of IGF-1 on day 3. ELISA showed that there was a significantly higher release of IGF-1 in a dose-dependent manner in the FO-treated cells (2555.9 ± 102.2, 2697.4 ± 73.7, and 3017.6 ± 268.3 pg/mL at 25, 50, and 100 µg/mL, respectively) than in the untreated cells (2161.1 ± 177.7 pg/mL). The amount of IGF-1 released at 100 µg/mL FO was comparable to that observed in the GP-treated cells (3043.6 ± 291.8 pg/mL). These results indicate that FO stimulated the expression of *IGF-1*, thereby enhancing the extracellular release of IGF-1.

### 2.4. Pharmacological Inhibition of IGF-1R Prevents FO-Induced Bone Formation and Growth Performance in Zebrafish Larvae

**Figure 4:**
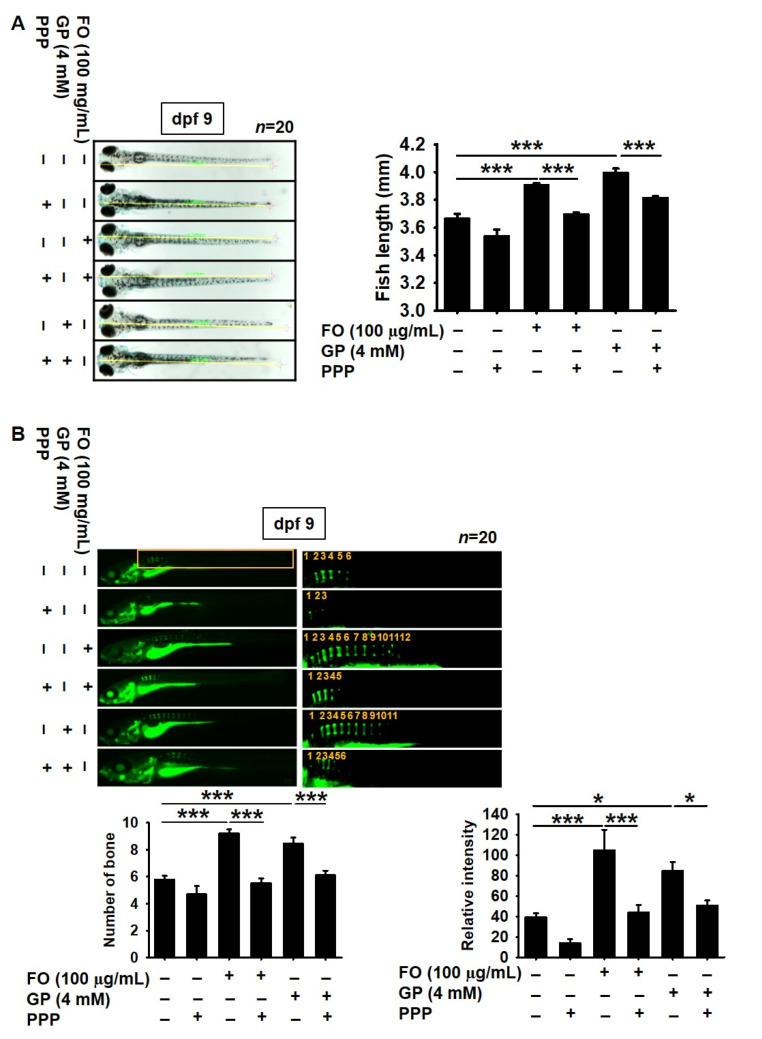
Pharmacological inhibition of IGF-1R decreases growth rate and bone formation in zebrafish larvae. Zebrafish larvae (*n* = 30) at 3 days post fertilization (dpf) were pretreated with 10 µM picropodophyllin (PPP) for 2 h prior to stimulation with 100 µg/mL FO or 4 mM β-glycerophosphate (GP). (**A**) Images of total body length were captured at 9 dpf (left) and the length is shown in the graph (right). (**B**) In a parallel experiment, the zebrafish larvae were stained with 0.05% calcein (top). Vertebrae number (bottom left) and relative density (bottom right) were calculated and are depicted in the graph. Significant differences among the groups were determined using one-way ANOVA followed by Bonferroni correction. All data are presented as mean ± SEM (* *p* < 0.05 and *** *p* < 0.001 vs. untreated zebrafish larvae). FO, fermented oyster (*C. gigas*) extract.

To address whether FO-induced IGF-1 triggers bone formation and growth rate, IGF-1R was pharmacologically inhibited by a cell permeable noncompetitive specific inhibitor, PPP, in zebrafish larvae at 3 dpf. A slight decrease in growth rate was observed in the PPP-treated zebrafish larvae at 9 dpf (3.53 ± 0.05 mm) than in the untreated larvae (3.66 ± 0.04 mm, Figure 4A). Additionally, pretreatment with PPP resulted in a significant retardation of total body growth from 3.91 ± 0.02 to 3.69 ± 0.01 mm in the FO-treated zebrafish larvae. GP-mediated increase in total body length also decreased in the presence of PPP, from 4.00 ± 0.03 to 3.81 ± 0.01 mm. We further evaluated whether FO induced vertebrae formation in zebrafish larvae through the IGF-1 signaling pathway. The total number of vertebrae in the PPP-treated larvae was lower than that in the untreated larvae (Figure 4B). Especially, 100 μg/mL FO-treated larvae showed a considerably higher number of vertebrae (9.19 ± 0.31, Figure 4B, bottom left) than the untreated zebrafish larvae (5.7 ± 0.28, Figure 4B, bottom left), which was concomitant with high bone calcification density (Figure 4B, bottom right). In addition, GP also enhanced vertebrae formation (8.45 ± 0.46, Figure 4B, bottom left) and calcein density (Figure 4B, bottom right) in zebrafish larvae. However, treatment with PPP caused a significant decrease in vertebrae formation (5.53 ± 0.35 and 6.11 ± 0.33, respectively) and calcification density induced by both FO and GP. These results indicate that FO enhanced bone formation-mediated growth performance.

### 2.5. FO Upregulates GSK-3β Phosphorylation at Ser9 and Thereby Promotes Nuclear Translocation of RUNX2, Leading to Calcium Deposition

**Figure 5:**
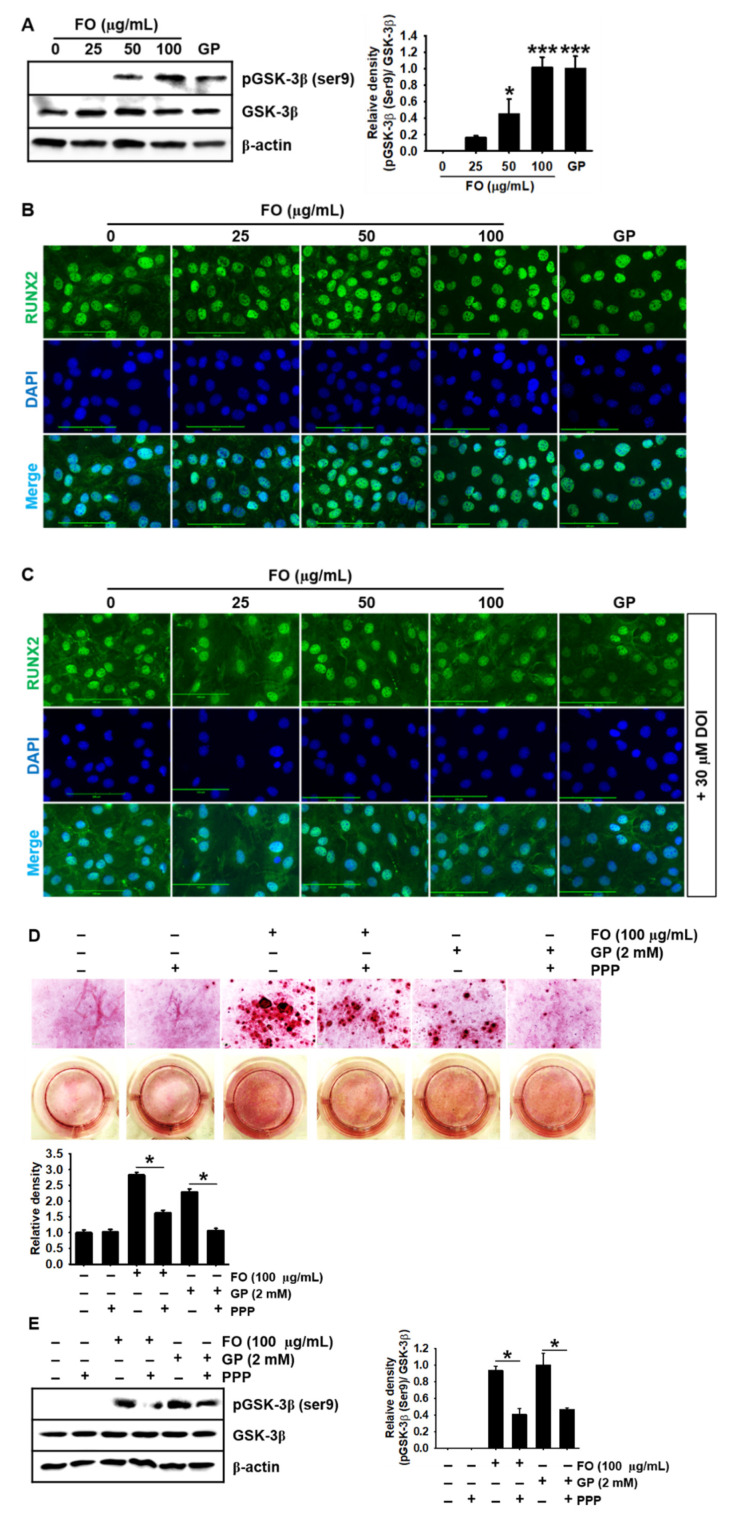
FO upregulates GSK-3β phosphorylation at Ser9 and thereby promotes the nuclear translocation of RUNX2, leading to calcium deposition. (**A**) MC3T3-E1 cells were treated with the indicated concentrations of FO (0–100 µg/mL) for 72 h. Total proteins were isolated and Western blotting was performed to measure the protein level of phosphorylated GSK-3β at Ser9. β-glycerophosphate (GP) at 2 mM was used as a positive control. (**B** and **C**) MC3T3-E1 cells were seeded on 3% gelatin-coated coverslips and pretreated with 30 µM DOI hydrochloride (DOI) for 2 h prior to treatment with FO or GP. The nuclear translocation of RUNX2 was measured by immunostaining, in the absence (**B**) and presence (**C**) of DOI. Scale bar = 100 µm. (**D**) MC3T3-E1 cells were seeded at a density of 2000 cells/cm^2^ and pretreated with 1 µM picropodophyllin (PPP) for 2 h prior to stimulation with 100 µg/mL FO and 2 mM GP. After 7 days, the cells were stained with alizarin red to determine the level of calcium deposition. (**E**) After 3 days in the presence of PPP, total proteins were isolated and Western blotting was performed to measure the level of phosphorylated GSK-3β at Ser9. Total GSK-3β was used as an internal control. The expression of phosphorylated GSK-3β relative to that of total GSK-3β is illustrated in the graphs. Significant differences among the groups were determined using the one-way ANOVA followed by Bonferroni correction. All data are presented as mean ± SEM (* *p* < 0.05 and *** *p* < 0.001 vs. untreated MC3T3-E1 cells). FO, fermented oyster (*C. gigas*) extract.

In our previous study, we found that FO significantly increased the nuclear translocation of β-catenin, resulting in the stimulation of bone formation [17]; however, we did not determine whether FO directly regulated GSK-3β. Therefore, in this study, we investigated whether FO induces GSK-3β phosphorylation at Ser9 (inactive form) and thus stimulates the nuclear translocation of RUNX2, a key regulator of bone formation and growth performance. As shown in Figure 5A, FO-treated cells showed a significant increase in GSK-3β phosphorylation at Ser9 in MC3T3-E1 cells, compared with the untreated cells. GSK-3β negatively regulates osteogenesis by inhibiting RUNX2 activity [16]; hence, we examined whether FO-induced GSK-3β phosphorylation at Ser9 promotes the nuclear translocation of RUNX2. In the untreated MC3T3-E1 cells, RUNX2 spontaneously moved into the nucleus; however, an enormous amount of RUNX2 was still present in the cytosol (Figure 5B). FO considerably triggered most RUNX2 to shift to the nucleus in a dose-dependent manner. In particular, the highest concentration of FO (100 μg/mL) strongly promoted the translocation of RUNX2 from the cytosol to the nucleus, which was comparable to the effect of GP. Interestingly, pretreatment with DOI, a potential stabilizer of GSK-3β mediated by 5-HT_2A_/5-HT_2C_ receptor, markedly downregulated the nuclear translocation of RUNX2 in the FO- or the GP-treated MC3T3-E1 cells (Figure 5C), indicating that FO-mediated GSK-3β inhibition is crucial for the nuclear translocation of RUNX2. Furthermore, we evaluated whether the IGF-1 signaling pathway would increase FO-induced calcium deposition in MC3T3-E1 cells by inhibiting GSK-3β. Alizarin red staining confirmed that FO and GP significantly increased calcium deposition; however, PPP, a pharmacological inhibitor of IGF-1R, impaired the deposition in MC3T3-E1 cells (Figure 5D). Finally, we found that FO- and GP-mediated GSK-3β phosphorylation at Ser9 was weakened in the presence of PPP, indicating that FO stimulated calcium deposition by inducing GSK-3β phosphorylation at Ser9 through the IGF-1R signaling pathway. Altogether, these results indicate that FO promoted bone mineralization by activating IGF-1-mediated RUNX2 through GSK-3β phosphorylation at Ser9.

### 2.6. Transient Knockdown of IGF-1Rα/β Decreases GSK-3β Phosphorylation at Ser9 in MC3T3-E1 Cells and Consequently Reduces Calcium Deposition

**Figure 6:**
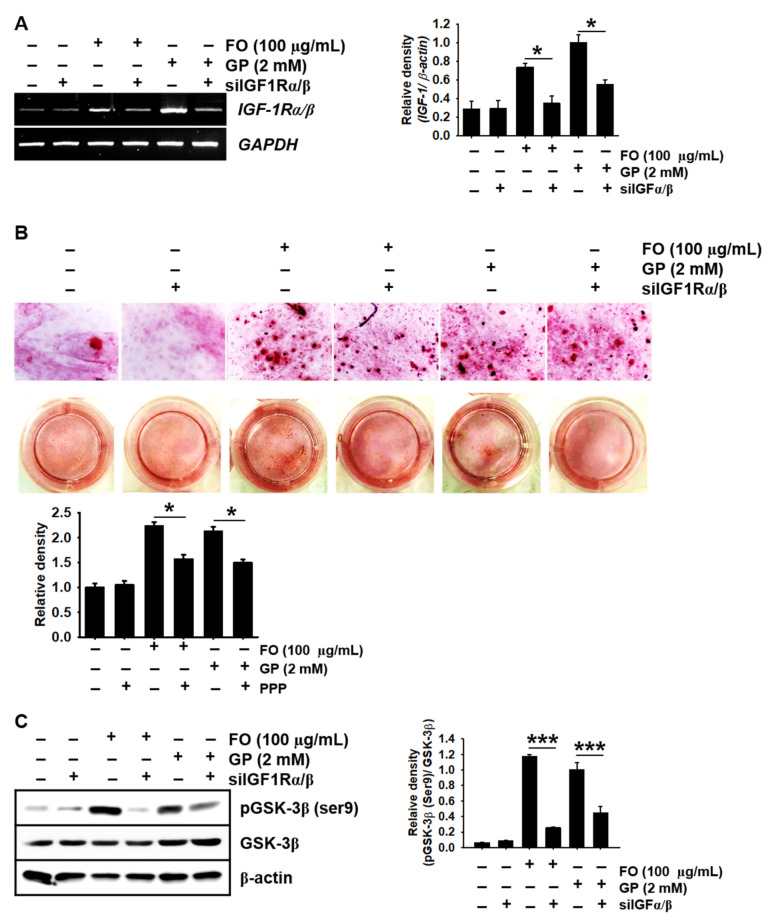
Transient knockdown of *IGF-1Rαβ* reduces calcium deposition concomitantly with a decrease in phosphorylated GSK-3β at Ser9. MC3T3-E1 cells were seeded at a density of 2000 cells/cm^2^ and transfected with silencing RNA for *IGF-1Rαβ* (siIGF-1Rαβ) 48 h before stimulation with 100 µg/mL FO or 2 mM GP. (**A**) Total mRNA was extracted, and reverse transcription polymerase chain reaction was performed. GAPDH was used as an internal control. (**B**) After 7 days, the cells were stained with alizarin red for calcium deposition, and images were captured. (**C**) Three days after treatment with FO, total proteins were isolated and Western blotting was performed to measure the protein level of phosphorylated GSK-3β at Ser9 (left). Total GSK-3β and β-actin were used as internal controls. The expression of *IGF-1Rαβ* and phosphorylated GSK-3β at Ser9 relative to *GAPDH* and total GSK-3β levels is illustrated (right). Significant differences among the groups were determined using one-way ANOVA followed by Bonferroni correction. All data are presented as mean ± SEM (* *p* < 0.05 and *** *p* < 0.001 vs. untreated MC3T3-E1 cells). FO, fermented oyster (*C. gigas*) extract.

Next, we confirmed whether the IGF-1R signaling pathway directly induced GSK-3β phosphorylation at Ser9 in FO-induced calcium deposition. Figure 6A shows that FO and GP significantly increased the expression of *IGF-1Rα*/*β* in MC3T3-E1 cells, and the transient knockdown of *IGF-1Rα*/*β* using siRNA significantly downregulated its expression. Consistent with the pharmacological inhibition of IGF-1R using PPP, siIGF-1Rα/β caused a significant decrease in alizarin red-stained calcium deposition in MC3T3-E1 cells under FO- and GP-treated conditions (Figure 6B). We further investigated whether GSK-3β phosphorylation at Ser9 was reduced under siIGF-1Rα/β-transfected conditions. Transfection with siIGF-1Rα/β resulted in the downregulation of phosphorylated GSK-3 at Ser9 in the presence of FO (relative density compared to GP-treated cells: 1.17 ± 0.02 and 0.26 ± 0.01 in the FO- and the FO + siIGF-1Rα/β-treated MC3T3-E1 cells and 1.00 ± 0.09 and 0.45 ± 0.08 in the GP- and the GP + siIGF-1Rα/β-treated MC3T3-E1 cells, respectively). Altogether, these results confirm that FO triggered IGF-1 expression and its binding to IGF-1R, which inhibited GSK-3β, stimulated RUNX2, and caused bone formation.

## 3. Discussion

**Figure 7:**
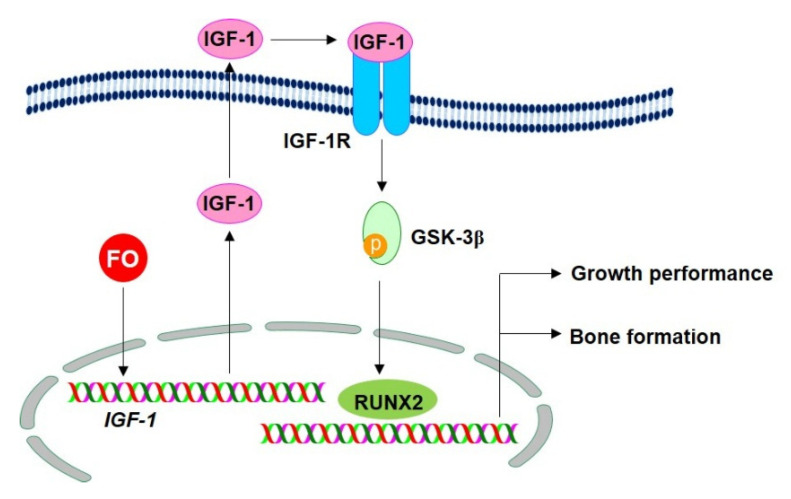
FO promotes bone formation and growth by activating the IGF-1/IGF-1R signaling pathway. FO upregulates growth-promoting genes such as insulin-like growth factor 1 (*IGF-1*), thereby promoting the release of IGF in osteoblast cells. The secreted IGF-1 binds to its specific receptor, IGF-1R, in an autocrine or paracrine manner and subsequently triggers the downstream signaling pathway. Once the IGF-1/IGF-1R complex is formed, it initiates the GSK-3β phosphorylation at Ser9. Phosphorylated GSK-3β subsequently releases RUNX2, which in turn promotes bone formation and growth. FO, fermented oyster (*C. gigas*) extract; IGF-1R, insulin-like growth factor-1 receptor; GSK-3β, glycogen synthase kinase-3β; RUNX2, runt-related transcription factor 2.

Pacific oysters (*C. gigas*) are popular edible shellfish which are consumed globally because of their abundant nutrients, vitamins, and minerals. Our previous studies confirmed that FO promotes osteoblast differentiation and bone formation by activating the β-catenin signaling pathway [17]. In addition, we found that the effect of FO was mediated by the inhibition of osteoclastogenesis through the downregulation of the receptor activator of nuclear factor kappa-B -induced generation of reactive oxygen species [18], which consequently prevented ovariectomy-induced bone loss [19]. Recently, Chen et al. also demonstrated that novel peptides isolated from *C. gigas* promoted osteoblast differentiation by binding to the integrin α/β [20]. Nevertheless, the underlying molecular mechanisms through which FO enhances growth performance based on bone formation remain unclear. In the current study, we found that FO accelerated bone formation and growth rate, which was accompanied by upregulated expression of growth-promoting genes. Especially, FO boosted IGF-1 release, which triggered the IGF-1R signaling pathway, subsequently phosphorylated GSK-3β at Ser9, and activated the nuclear translocation of RUNX2, leading to bone formation and growth performance. In addition, pharmacological and siRNA-based inhibition of IGF-1R restrained FO-induced bone formation and growth rate. In this regard, FO may have the potential to promote bone formation and growth through the stimulation of the IGF-1R signaling pathway (Figure 7). However, the mechanism through which FO stimulates the expression of *IGF-1* still needs to be evaluated.

Cholecystokinin a (*zccka*), an ortholog for the mammalian *cck*, is a peptide hormone secreted by cells in the duodenum in zebrafish [21]. It improves growth performance by stimulating the digestion of fat and protein [22]. Glucokinase (*zgck)* is another growth-promoting enzyme in zebrafish and an ortholog of human *gck* [23]; it is involved in glucose metabolism by facilitating the phosphorylation of glucose in glucose-6-phosphate, leading to an increase in growth performance [24]. In spite of its role in glucose metabolism, glucokinase is also needed for pancreatic regeneration and type B pancreatic cell development [25]. Zebrafish growth hormone 1 (*zgh1*) is an ortholog of mammalian *gh*, and it is involved in the production of growth hormone. Growth hormone binds with its cognitive receptors such as GH-R1 [26] and GH-Ra [27], which are encoded by *gr1-1* and *ghra*, respectively. It is involved in the development of the adipose tissue and the positive regulation of the lipid catabolism process [28]. As previously mentioned, growth performance is related to the rate at which nutrients such as carbohydrates, lipids, and proteins are absorbed in the intestine. In addition, the rate at which growth hormones transfer their stimulating signals in the cells through their specific receptors is very important. In the current study, we found that FO enhanced the growth rate of zebrafish larvae, which was accompanied by increased expression of growth-promoting genes such as *zIGF-1*, *zIGFBP-3*, *zgh-1*, *zghr-1*, *zghra*, *zgck*, and *ccka*. Nevertheless, we still do not know the involvement of these genes in FO-induced bone formation and growth performance.

IGF-1 is primarily produced by the liver as an endocrine hormone and by some tissues as an autocrine/paracrine hormone. It is considered essential for longitudinal bone growth, skeletal maturation, and bone mass acquisition during growth performance and maintenance, whereas lower serum IGF-1 levels are associated with the prevalence of fractures and increased risk of osteoporosis in postmenopausal women [29]. Therefore, serum IGF-1 has been used as a potential indicator for biochemical growth maturity [30] and accelerated linear growth rate in clinical trials [1]. Guerra-Menendez et al. showed that heterozygous IGF-1 mice (*IGF-1^+^*^/*−*^) had a reduction in bone density accompanied by low levels of RUNX2 activity, whereas treatment with IGF-1 led to a significant elevation of bone mass and RUNX2 expression, which indicates that IGF-1 promotes osteogenesis by activating RUNX2 [31]. Additionally, IGF-1 stimulates systemic body growth by enhancing growth-promoting properties in most cells, including osteoblasts and muscle cells [32]. In the current study, we found that FO increased growth rate in zebrafish larvae, accompanied by IGF-1 release and the inhibition of the IGF-1R signaling pathway. PPP strongly inhibited FO-induced bone formation and growth rate, indicating that FO-mediated bone formation and growth performance require the presence of IGF-1. Recently, Werner et al. reported that pituitary-produced GH leads to the secretion of IGF-1 by the liver into the systemic circulation, which causes bone elongation and longitudinal growth [33]. Mice lacking GH and IGF-1 receptor (*Gh^−^*^/*−*^ and *IGF-1^−^*^/*−*^) showed greater reduction in bone mass and length compared with the wild-type (*Ghr^+^*^/*+*^ and *IGF-1^+^*^/*+*^) mice, suggesting that GH and IGF-1 are important for bone formation [34]. In the current study, we also found that FO increased the expression of *GH*, and GH may be involved with IGF-1 in bone formation and growth performance. Nevertheless, the mechanism through which FO regulates the joint involvement of IGF-1 and GH in bone formation and growth performance was not studied in the current study. Therefore, further studies should be conducted to determine if the involvement of GH is closely related to that of IGF-1 in FO-induced bone formation and growth performance.

IGF-1 directly binds to its specific receptor, IGF-1R, on the cell surface and activates receptor tyrosine kinase in IGF-1R, leading to osteogenesis and growth performance [35]. In this regard, we found that FO stimulated the release of IGF-1 and expression of IGF-1R, which triggered the IGF-1R signaling pathway through the autocrine effect of IGF-1, resulting in an increase in osteogenesis-mediated growth performance. When IGF-1 does not bind to IGF-1R on the cell membrane surface, IGF-1R shuts down its cellular signals by switching off the PI3K/Akt pathway, which induces non-phosphorylated GSK-3β at Ser9 and consequently inhibits RUNX2 and β-catenin. Previously, our research demonstrated that FO increased osteoblast differentiation by upregulating the nuclear translocation of active β-catenin, resulting in bone formation [17]. However, the molecular mechanism associated with the release of β-catenin from its destructive complex has not been elucidated. In this study, FO-induced IGF-1 acted as an autocrine hormone for bone formation and growth performance by inducing GSK-3β phosphorylation at Ser9, suggesting that RUNX2 and β-catenin were released from GSK-3β. Consistent with our findings, Wang et al. showed that IGF-1/IGF-1R promoted the Wnt/β-catenin signaling pathway and pharmacological inhibition of IGF-1R by PPP resulted in the lowering of β-catenin levels, suggesting that IGF-1 plays a crucial role in the activation of β-catenin in osteoblast differentiation [36]. RUNX2 is also a vital transcription factor for the maintenance of bone homeostasis and growth performance. Homozygous *RUNX^−/−^* adult mice were associated with reduced trabecular bone volume, cortical thickness, bone mineral density, and greater suppression of osteoblastic markers compared to their wild-type counterparts [37]. Aligned with β-catenin, endogenous RUNX2 activity is mainly regulated by GSK-3β, but via a different manner [6]. We found that FO promoted bone formation and growth by upregulating growth-promoting genes through the activation of the IGF-1-GSK-3β- RUNX2-mediated signaling pathway. Nevertheless, many other factors such as TWISTs and FOXOs inhibit RUNX2 activity; hence, FO-induced RUNX2 regulation should be thoroughly investigated [38,39].

In conclusion, FO considerably promoted bone formation and growth performance by increasing the expression and release of autocrine IGF-1 from osteoblast cells, which triggered the IGF-1R signaling pathway. Meanwhile, FO inactivated GSK-3β through GSK-3β phosphorylation at Ser9 and consequently stimulated bone- and growth-promoting transcription factors such as β-catenin and RUNX2. Therefore, FO is a potential food supplement for bone formation and improved growth performance.

## 4. Materials and Methods

### 4.1. Reagents and Antibody

First, 3-(4,5-Dimethylthiazol-2-yl)-2,5-diphenyl-tetrazolium bromide (MTT), calcein, alizarin red, gelatin, donkey serum, 4′6-diamidino-2-phenylindole (DAPI), and β-glycerophosphate (GP) were purchased from Sigma (St. Louis, MO, USA). Minimum essential medium alpha modification (α-MEM), fetal bovine serum (FBS), and antibiotic mixtures were obtained from WelGENE (Gyeongsan, Gyeongsangbuk-do, Republic of Korea). Antibodies against GSK-3β (sc-81462), phospho-GSK-3β at Ser9 (sc-37800), RUNX2 (sc-101145) and peroxidase-labeled anti-mouse immunoglobulins (sc-516102) were purchased from Santa Cruz Biotechnology (Santa Cruz, CA, USA). Peroxidase-labeled anti-rabbit immunoglobulins (KO211708) were obtained from KOMA BIOTECH (Seoul, Republic of Korea). Picropodophyllin (PPP) and DOI hydrochloride (DOI) were obtained from Tocris (Bristol, UK). Alexa Fluor^®^ 488 goat anti-rabbit secondary antibody was purchased from Abcam (Cambridge, UK). Dako faramount aqueous mounting medium was purchased from Dako (Carpinteria, CA, USA). Commercial FO (product name: FO100) was kindly supplied by Marine Bioprocess Co. (Busan, Republic of Korea). FO contains approximately 60% protein, 30% carbohydrate, and 3% lipid, as previously described [17]. All other chemicals were purchased from Sigma.

### 4.2. Cell Culture

Mouse preosteoblast MC3T3-E1 cells were obtained from American Type Culture Collection (ATCC, Manassas, VA, USA) and cultured at 37 °C in 5% CO_2_ in α-MEM supplemented with 10% FBS.

### 4.3. Isolation of Total RNA from Mouse Preosteoblast MC3T3-E1 Cells and Reverse Transcription Polymerase Chain Reaction (RT-PCR)

MC3T3-E1 cells were cultured in 24-well plate at a density of 2000 cells/cm^2^ overnight and then treated with FO (0–100 µg/mL) or GP (2 mM) for 5 days. Total RNA was extracted using an easy-BLUE^TM^ RNA extraction kit (iNtRON Biotechnology, Seongnam, Gyeonggi-do, Republic of Korea), according to the manufacturer’s instructions. Genes of interest were reverse-transcribed and amplified using One-Step RT-PCR Premix (iNtRON Biotechnology). The specific primers for *mIGF-1* (sc-37194-PR) and *mIGF-1Rα/β* (sc-35638-PR) were purchased from Santa Cruz Biotechnology (Santa Cruz, CA). PCR conditions were provided according to the manufacturer protocol. Mouse glyceraldehyde 3-phosphate dehydrogenase (*GAPDH)* (F: 5′-CACCACCCTGTTGCTGTAGC-3′ and R: 5′-ACCACAGTCCATGCCATCAC-3′) was used as an internal control to evaluate the relative expression of *mIGF-1* and *mIGF-1Rα/β*.

### 4.4. Measurement of IGF-1by Enzyme-Linked Immunosorbent Assay (ELISA)

MC3T3-E1 cells were cultured in 24-well plate at a density of 2000 cells/cm^2^ overnight. Then, the cells were pretreated with the indicated concentrations of FO (0–100 µg/mL) or GP (2 mM) for 72 h, and cell free supernatants were collected. The supernatants were assayed for the concentration of IGF-1 using an IGF-1 ELISA kit (K033225, KOMA BIOTECH) according to the manufacturer’s protocol.

### 4.5. Western Blot Analysis

Total protein extracts were prepared using radio immunoprecipitation assay (RIPA) lysis buffer (iNtRON Biotechnology). Lysates were centrifuged at 14,000× *g* at 4 °C for 20 min. Supernatants were collected and stored at −80 °C until further use. Protein concentrations were measured using a Bio-Rad protein assay kit (Bio-Rad, Hercules, CA, USA). Equal amount of protein was separated on SDS-polyacrylamide gels, transferred to PVDF membrane (Thermo Fisher Scientific, Sunnyvale, CA, USA), and immunoblotted with the indicated antibodies. Bound antibodies were detected using an enhanced chemiluminescence plus kit (Thermo Fisher Scientific, Sunnyvale, CA, USA). The images were captured by ImageQuant LAS 500 (GE Healthcare Bio-Sciences AB, Uppsala, Sweden). The expressional values of proteins were normalized to the level of total GSK-3β.

### 4.6. Immunostaining of RUNX2

MC3T3-E1 cells (2000 cells/cm^2^) were seeded on 3% gelatin-coated coverslips and allowed to attach in cover slips. Then, FO (0–100 µg/mL) was treated in the presence or absence of 30 µM DOI. After 3 days, the cells were fixed with 4% paraformaldehyde for 10 min at 37 °C and washed three times with ice-cold phosphate buffered saline (PBS). The fixed cells were permeabilized with 0.1% Triton X-100 for 10 min at room temperature. After washing with ice-cold PBS containing 0.1% tween-20 (PBST) for 5 min, the cells were blocked with 10% donkey serum and incubated with RUNX2 antibody (1:100 in 10% donkey serum) overnight at 4 °C. The cells were stained with Alexa Fluor^®^ 488 secondary antibody for 2 h and counterstained with a nuclear staining die, DAPI (300 nM), for 10 min. After being washed three times with ice-cold PBST, the coverslips were mounted onto glass slides with Dako faramount aqueous mounting media and fluorescence images were captured by CELENA^®^ S digital imaging system (Logos Biosystems, Anyang, Gyeonggi-do, Republic of Korea).

### 4.7. Transient Knockdown of IGF-1Rα/β

MC3T3-E1 cells were transfected with silencing RNA for *IGF-1Rα*/*β* (siIGF-1Rα/β (sc-35638), Santa Cruz). Briefly, the cells were seeded at a density of 2000 cells/cm^2^ in 6-well cell culture plates overnight and replaced with FBS-free media. Serum free growth medium was added to 10 nM siIGF-1Rα/β with the transfection reagent, G-Fectin (Genolution Pharmaceuticals Inc., Seoul, Republic of Korea), and the entire mixture was incubated for 15 min at room temperature and gently added to the cells. The cells were kept for 48 h to facilitate the transfection before being subjected to the subsequent treatments.

### 4.8. Alizarin Red Staining

MC3T3-E1 cells were seeded in a 24-well plate at a density 2000 cells/cm^2^ and then treated with the indicated concentrations of FO (0–100 µg/mL). In vitro calcium deposition was measured by staining with alizarin red. Briefly, MC3T3-E1 cells were washed with PBS and fixed with 4% paraformaldehyde for 30 min at 37 °C. Then, the cells were washed with PBS and stained with 2% alizarin red solution for 30 min. Images of each well were captured with a phase contrast microscope (Ezscope i900PH, Macrotech, Goyang, Gyeonggi-do, Republic of Korea).

### 4.9. Maintenance of Zebrafish Embryo and Larvae

Zebrafish (AB strain) were maintained and raised according to standard guidelines of the Animal Care and Use Committee of Jeju National University (Jeju, Jeju Special Self-Governing Province, Republic of Korea; approval no.: 2020-0029). The adult zebrafish were raised at 28.5 °C with a 14:10-h light:dark cycle. Fertilized embryos after natural spawning were collected and cultured at 28.5 °C in E3 embryo media containing 2 mg/L methylene blue.

### 4.10. Measurement of Total Body Length in Zebrafish Larvae

In order to measure growth rate, zebrafish larvae at 3 days post fertilization (dpf) were treated with 0–100 µg/mL FO or 4 mM GP. Media were replaced with FO or GP every 3 day. Total body length was measured at 6, 9, and 12 dpf using an Olympus SZ2-ILST stereomicroscope (Tokyo, Japan). In a parallel experiment, 3 dpf zebrafish larvae were pretreated with PPP for 2 h prior to stimulation with 100 µg/mL FO or 4 mM GP. Media were replaced at 6 dpf with FO or GP, and total body length of zebrafish larvae was measured at 9 dpf.

### 4.11. Bone Formation in Zebrafish Larvae

In order to monitor bone formation in zebrafish larvae, calcein green fluorescent marker was used. To analyze vertebrae formation, zebrafish larvae at 3 dpf were treated with 0–100 µg/mL FO and media were replaced at 6 dpf with FO. At 9 dpf, the larvae were immersed in 0.05% calcein solution for 10 min and then rinsed in fresh water three times for 10 min. The larvae were anesthetized in 0.04% tricaine methanesulfonate solution and mounted on depression slides using 2% methylcellulose. Fluorescence images were captured by a CELENA^®^ S digital imaging system (Logos Biosystems, Anyang, Gyeonggi-do, Republic of Korea).

### 4.12. Isolation of Total Zebrafish RNA and RT-PCR

Zebrafish larvae at 3 dpf were treated with 0–100 µg/mL FO or 4 mM GP, and total RNA was extracted at 9 and 12 dpf using an easy-BLUE^TM^ total RNA extraction kit. The RNA was reverse-transcribed by an MMLV reverse transcriptase kit (BIONEER, Daejeon, Republic of Korea) and synthetic cDNA was amplified using following primers (Table 1).

### 4.13. Statistical Analysis

All Western blots, RT-PCR bands, and zebrafish vertebrae were quantified by ImageJ 1.50i (National Institute of Health, Manassas, VA, USA) and then statistically analyzed by Sigma plot 12.0. All data represent the mean of at least three independent experiments and significant differences between groups were determined using an unpaired one-way ANOVA test with Bonferroni correction. Statistical significance was set at * *p* < 0.05, ** *p* < 0.01, and *** *p* < 0.001.

## Figures and Tables

**Table 1:** Zebrafish primers and PCR conditions used in this experiment.

Gene *	Primer Sequences (5′→3′)	Amplicon (bp)	T_m_	Cycle No.
*zccka*	F: GAT GAA GAA CCT CGC AGC AG	154 bp	58 °C	27
	R: GGC CCA AAT CCA TCC ATC CC			
*zghr-1*	F: TCA GTC CGA CTC AGA AAC CG	178 bp	58 °C	27
	R: TTC TGA AGC ACG GGA CCA TA			
*zgck*	F: GTA GGT ACA GGC TGC AAT GC	224 bp	58 °C	27
	R: TCA CCC CTG TAC TTC CCA CC			
*zgh-1*	F: GGT GGT ACA GGC TGC AAT GC	157 bp	58 °C	27
	R: CAA CTGTCT GCG TTC CTC AG			
*zghra*	F: CAT TGT CAT TCC CCA GCA GC	214 bp	58 °C	27
	R: ATC TGC AGG ATC GTC GAT GT			
*Zigf-1*	F: GAG TAC CCA CAC CCT CTC AC	213 bp	58 °C	27
	R: TGA AAG CAG CAT TCG TCC AC			
*ighfbp3*	F: AGG ACA CCA TCA GAA CCC AG	182 bp	58 °C	27
	R: CGA CGA CAT GGG CCA TAT TC			
*zβactin*	F: CGA GCG TGG CTA CAG CTT CA	155 bp	61 °C	27
	R: GAC CGT CAG GCA GCT CAT AG			

bp: base pair, T_m_: melting temperature, * *zigf-1*: insulin-like growth factor-1, *zigfbp-3*: insulin-like growth factor binding protein 3, *zgh-1*: growth hormone-1, *zghr-1*: growth hormone receptor-1, *zghra*: growth hormone receptor alpha, *zgck*: glucokinase, and *zccka*: cholecystokinin.
